# *Petasites japonicus* Stimulates the Proliferation of Mouse Spermatogonial Stem Cells

**DOI:** 10.1371/journal.pone.0133077

**Published:** 2015-07-24

**Authors:** Hye-Ryun Kang, Yong-An Lee, Yong-Hee Kim, Dong Gu Lee, Bang-Jin Kim, Ki-Jung Kim, Byung-Gak Kim, Myeong-Geun Oh, Chan Kyu Han, Sanghyun Lee, Buom-Yong Ryu

**Affiliations:** 1 Department of Animal Science and Technology, Chung-Ang University, Anseong, Republic of Korea; 2 Department of Integrative Plant Science, Chung-Ang University, Anseong, Republic of Korea; 3 Department of Obstetrics, Gynecology & Reproductive Biology, Van Andel Institute, Michigan State University, Grand Rapids, Michigan, United States of America; 4 Korea Food Research Institute, Sungnam, Republic of Korea; Rutgers - New Jersey Medical School, UNITED STATES

## Abstract

Oriental natural plants have been used as medical herbs for the treatment of various diseases for over 2,000 years. In this study, we evaluated the effect of several natural plants on the preservation of male fertility by assessing the ability of plant extracts to stimulate spermatogonial stem cell (SSC) proliferation by using a serum-free culture method. *In vitro* assays showed that *Petasites japonicus* extracts, especially the butanol fraction, have a significant effect on germ cells proliferation including SSCs. The activity of SSCs cultured in the presence of the *Petasites japonicus* butanol fraction was confirmed by normal colony formation and spermatogenesis following germ cell transplantation of the treated SSCs. Our findings could lead to the discovery of novel factors that activate SSCs and could be useful for the development of technologies for the prevention of male infertility.

## Introduction

Spermatogenesis is a highly coordinated, multistep process involving male germ cell proliferation and differentiation. It starts with sequential mitotic cell divisions of spermatogonia, followed by meiosis of spermatocytes to form round spermatids [1]. Spermatogonial stem cells (SSCs) are the adult stem cells that are capable of both self-renewal and differentiation into daughter spermatogonia. This dual capacity ensures that the testes produce spermatozoa throughout life [2]. The essential functions of SSCs in adult male fertility have been well recognized and have been globally investigated to determine the underlying regulatory mechanism. In addition, methods for the isolation, culture, and transplantation of SSCs have facilitated the development of clinical applications for preserving human male fertility [3–6].

Decreased tissue regenerative potential is a hallmark of aging, and the regenerative potential of tissue-specific stem cells can be modulated by systemic factors that change with age. This age-related impairment may be due to increased oxidative damage, mitochondrial dysfunction, endocrine imbalance, or a lack of paracrine factors [7]. Ryu and colleagues reported that the declining function of Sertoli cells is a major factor in age-related male infertility [8]. Growing evidence suggests that male reproductive function declines with age and that spermatogenesis exhibits age-related deficits, ultimately resulting in infertility [8,9]. Because aging is a phenomenon that affects all self-renewing tissues in the body and may result from defects in the local microenvironment, studies aimed at prolonging male fertility are needed in the fields of clinical and preventive medicine [8]. Recently, a few studies have investigated various plant extracts that may modulate the proliferation of many tissue and cell types, including insulinoma, skin, bone marrow, and hematopoietic cells [10–15]. Other studies have shown that dietary fatty acids, particularly oleic acid and linolenic acid, actively promote the proliferation of hematopoietic stem cells [10,16,17] and modulate the self-renewal of intestinal epithelial cells [18]. The potential of natural plant extracts to support the growth of many cell types has been confirmed as reported above. It appears that natural materials could be a useful resource for studies on the regulation of the proliferation and differentiation of SSCs.

The purpose of this study was to identify useful natural plant sources and investigate the properties of various natural plant-derived extracts for their ability to stimulate the proliferation of SSCs and maintain their stem cell activity. By adding various plant extracts to the serum-free medium of SSC cultures, we showed for the first time, to the best of our knowledge, that a butanol fraction of *Petasites japonicus* (*P*. *japonicus*) can promote SSC proliferation. We also demonstrated that SSCs recovered from cultures treated with the butanol fraction of *P*. *japonicus* were able to colonize recipient testes. The findings of this study are expected to serve as a foundation for research on the control of SSC proliferation and differentiation and the development of new medicines for the treatment of male infertility.

## Materials and Methods

### Plant materials and extraction

**Fig 1:**
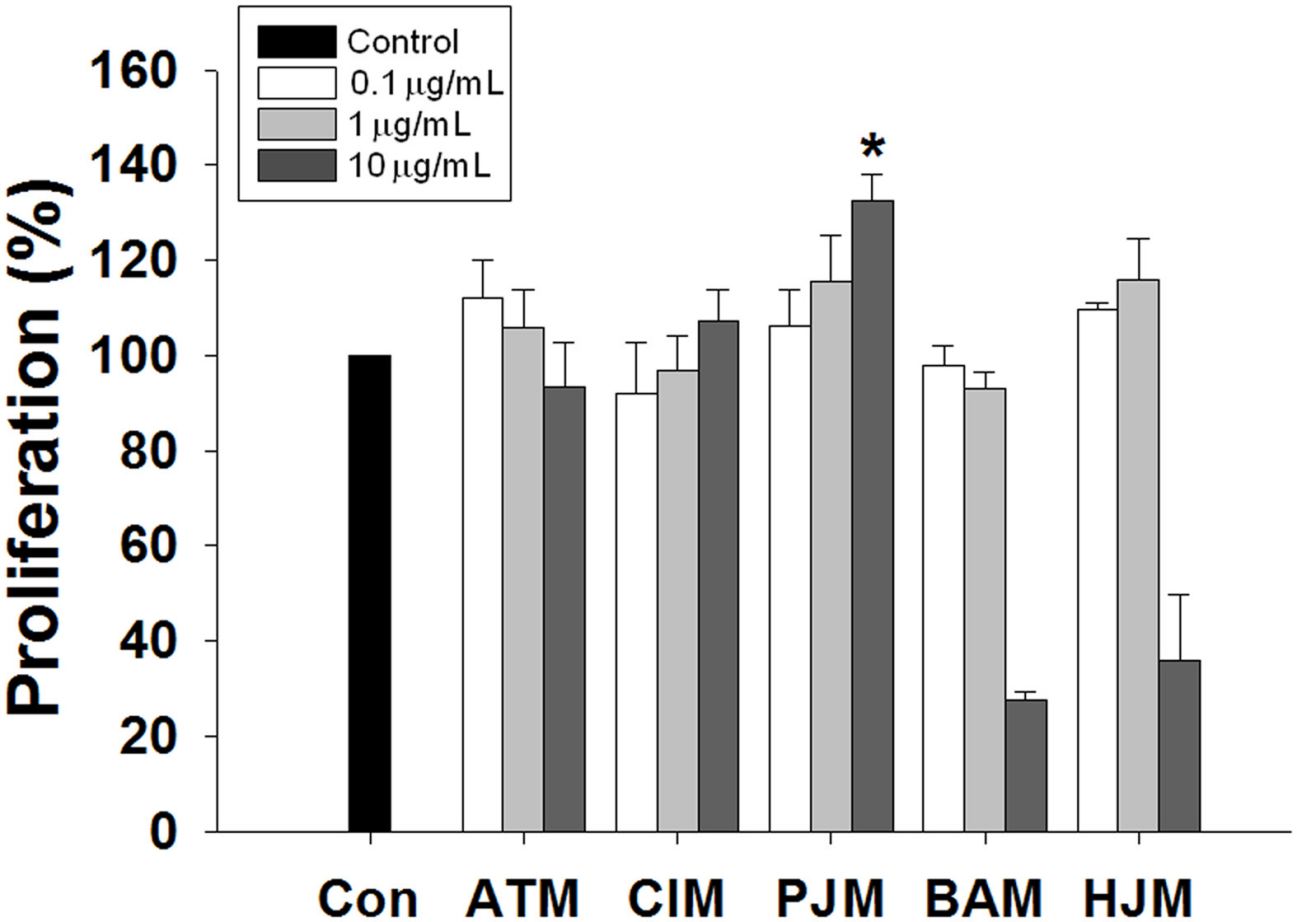
The effect of plant-derived extracts on SSC proliferation activity. To assess the proliferation-promoting effects of various natural plant-derived extracts on SSCs in culture, the number of germ cells was determined after seven days of culture in serum-free medium containing GDNF without any plant extract (control) or with different concentrations (0.1, 1, and 10 μg/mL) of various plant extracts. Values are means ± SEM (n = 3 independently established cultures for each treatment). Asterisks indicate a significant increase (*P* < 0.05) compared to the control. Con, control; ATM, *Aster tataricus*; CIM, *Chrysanthemum indicum*; PJM, *Petasites japonicus*; BAM, *Berberis amurensis* var. *latifolia*; HJM, *Humulus japonicus*.

Unless otherwise stated, all reagents were purchased from Sigma-Aldrich (St. Louis, MO, USA). We prepared extracts from various plants, including *Aster tataricus*, *Chrysanthemum indicum*, *P*. *japonicus*, *Berberis amurensis* var. *latifolia*, and *Humulus japonicus*, that have either been used ethnobotanically or are taxonomically closely related to traditional botanical medicines [19–23]. All specimens were botanically authenticated by Prof. Seon Haeng Cho, at Gongju National University of Education, Republic of Korea. Voucher specimens were deposited in the Herbarium at the Department of Integrative Plant Science, Chung-Ang University, Republic of Korea. Fresh plant leaves were cleaned and rinsed with water. The leaves were air-dried, ground into powder, and extracted with methanol (MeOH) under reflux at 70°C for 3 h. The filtrate was concentrated *in vacuo* until dry to produce a MeOH extract, which was suspended in dimethyl sulfoxide (DMSO) for further analysis. All plant extracts were added to the cell cultures as described in the Results section. Based on the results of the screening data (Fig 1), we selected the MeOH extract of *P*. *japonicus* (PJM) for sequential fractionation studies.

### *P*. *japonicus* fractionation

PJM was fractionated with *n*-hexane, methylene chloride (MC), ethyl acetate (EtOAc), or *n*-butanol (*n*-BuOH). Briefly, the dried powder was mixed with 300 mL of solvent and was stirred on a hot plate at 40°C. After 3 h, the fraction was filtered, and the resulting residue was re-extracted twice with the same solvent. The extraction process was continued using solvents of increasing polarity; each extraction was performed thrice using the same solvent. The *n*-hexane, MC, EtOAc, and *n*-BuOH fractions were pooled and evaporated using a rotary evaporator (EYELA N-1000; Rikakikai Co., Tokyo, Japan). The extractions were subsequently dissolved in 10% DMSO and stored at -20°C until further analysis.

### Experimental animals

All animal procedures were approved by the Animal Care and Use Committee of Chung-Ang University (Permit Number: 14–0072) and were performed in accordance with the *Guide for the Care and Use of Laboratory Animals* published the National Institutes of Health. Animals were housed under semi-barrier conditions at 21 ± 2°C, 55 ± 10% humidity, and a 12:12 light:dark cycle (with lights on at 0700 and lights off at 1900). C57BL/6-TG-EGFP (designated C57GFP) mice that express enhanced green fluorescent protein (EGFP) in all cells, which allows for the identification of donor cells in non-EGFP recipients, were purchased from Jackson Laboratory (Bar Harbor, ME, USA). C57BL/6 mice, obtained from Harlan Laboratories (Indianapolis, IN, USA), were used as the recipients in the SSC quantification experiments. A completed ARRIVE guidelines checklist is included in S1 Checklist.

### Isolation and *in vitro* culture of undifferentiated germ cell populations enriched for SSCs

Undifferentiated germ cell populations containing SSCs were obtained from 6- to 8-day-old C57GFP mice, and cell populations enriched for SSCs were isolated as previously described with minor modifications [24]. Briefly, seminiferous tubules were obtained from fresh testes by decapsulation and placed in Dulbecco’s Phosphate Buffered Saline (DPBS; Life Technologies, Grand Island, NY, USA). The tubules were enzymatically digested by incubation in a 2:1 solution of 0.25% trypsin-EDTA (Life Technologies) and 7 mg/mL DNase I (Roche, Basel, Switzerland) in DPBS at 37°C for 5 min. Enzymes were inactivated by the addition of 10% (final volume) fetal bovine serum (FBS; Thermo Fisher Scientific, Waltham, MA, USA). Testis cell suspensions were then filtered through a nylon mesh with 40-μm pores (BD Biosciences, San Jose, CA, USA), and cell viability was determined by trypan blue exclusion. Viability after digestion and filtration was ≥95%. After filtration, cells were centrifuged at 600 × *g* for 7 min at 4°C, and the pellet was resuspended at a density of 5 × 10^6^ cells/mL in Dulbecco’s modified Eagle medium (DMEM; Life Technologies) containing 10% FBS, 2 mM L-glutamine, 0.1 mM β-mercaptoethanol, 100 U/mL penicillin, and 100 μg/mL streptomycin. To remove erythrocytes and cellular debris from the testis cells, 10 × 10^6^ cells were overlaid on 2 mL of a 30% Percoll solution and centrifuged. The resulting pellet was resuspended, and Thy-1-positive (Thy-1^+^) cells, which were enriched for SSCs, were isolated by magnetic-activated cell sorting (MACS) using anti-Thy-1 antibody microbeads (Miltenyi Biotech, Auburn, CA, USA). The isolated Thy-1^+^ cells were seeded at a density of 0.1 × 10^6^ cells per well in 12-well culture plates containing mitotically inactivated SIM mouse embryo-derived thioguanine- and ouabain-resistant (STO) feeder cells. The cultures were maintained in mouse serum-free medium (mSFM) containing 10 ng/mL glial cell line-derived neurotrophic factor (GDNF; R&D Systems, Minneapolis, MN, USA), 75 ng/mL GDNF family receptor α1 (GFRα1; R&D Systems), and 1 ng/mL basic fibroblast growth factor 2 (bFGF2; BD Biosciences, San Jose, CA, USA) as previously reported [25]. Cell cultures were passaged 1:2 or 1:3 every seven days, and the medium was replaced every 2–3 days.

### Cell proliferation analysis

For the cell proliferation assay, germ cells enriched for SSCs (9–16 passages) were cultured for seven days in 24-well plates at a density of 2 × 10^5^ cells per well as described above. The cells were treated with the various natural plant extracts at concentrations of 0.1, 1, and 10 μg/mL, and the culture medium containing each extract was replaced every two days. The cells were detached from the culture plate by digestion with 0.25% trypsin-EDTA for 3 min, and the differences in cell proliferation between treatment groups were evaluated by counting the number of cells after seven days of culture and normalizing it to the number of cells cultured without treatment (control) using the following equation:

Normalized data (%) = number of recovered cells after culture × 100∕number of recovered control cells after 7 days of culture

### Immunocytochemistry

After 7 days of culture, germ cells enriched for SSCs were fixed with 4% paraformaldehyde for 20 min and permeabilized with 0.1% Triton X-100 in DPBS for 10 min at room temperature. After permeabilization, the cells were blocked with 5% (w/v) bovine serum albumin (BSA) in DPBS for 30 min to avoid nonspecific antibody binding, and then incubated with a mouse anti-human promyelocytic leukemia zinc finger (PLZF, 1:200; EMD Millipore, Billerica, MA, USA) primary antibody at 4°C overnight. The following day, the cells were washed thrice in DPBS and incubated with an Alexa Fluor 488-conjugated goat anti-mouse IgG secondary antibody (Life Technologies) for 1 h at room temperature. Labeled cells were washed thrice in DPBS, mounted with VectaShield mounting media containing DAPI, and analyzed using a Nikon TS-1000 microscope with NIS Elements imaging software (Nikon, Chiyoda-ku, Tokyo, Japan).

### Quantitative real-time PCR (qRT-PCR) assay

Total RNA was prepared and real-time RT-PCR using SYBR Green PCR master mix (Life Technologies) was performed according to the manufacturer’s instructions. The primers used are shown in Table 1. The relative mRNA expression of each gene was normalized to the level of glyceraldehyde-3-phosphate dehydrogenase (*GAPDH*) mRNA. The data are presented as mean ± SEM from three independent experiments performed in triplicate.

**Table 1:** Primers used for qRT-PCR.

Gene	Forward Primer (5′–3′)	Reverse Primer (5′–3′)
*LHX1*	aggagccccgagattactgt	ctcgggtaccacgcaagtat
*GFRα1*	ccactcctggatttgctgat	gctgaagttggtttccttgc

### Germ cell transplantation

A germ cell transplantation experiment was conducted to directly evaluate the effects of the natural plant-derived extracts on the SSC population. The only method available to definitively quantify the number of SSCs in a given cell population is the germ cell transplantation assay [26,27]. C57 mice were treated with 44 mg/kg body weight (b.w.) busulfan at six weeks of age to deplete endogenous germ cells and were then used as recipients for the C57GFP donor cell transplantation. After recipient preparation, germ cells recovered from the culture treated with the experimental plant extracts for seven days were concentrated to a density of 5 × 10^6^ cells/mL and stained with trypan blue for visualization during injection. Recipient mice were anesthetized intraperitoneally (i.p.) with 75 mg/kg b.w. ketamine and 0.5 mg/kg b.w. medetomidine, and donor cells were injected into the testes through the efferent ducts as previously described [26,27]. Approximately 8 μL of the donor cell suspension was introduced into the testes of the recipients, resulting in 80% filling of the seminiferous tubules.

### Analysis of stem cell activity and fertility

Two months after transplantation, the recipients were euthanized, and the testes were recovered and decapsulated. To quantify the SSCs, the number of fluorescent donor colonies longer than 1 mm in length was counted in each recipient testis using fluorescence microscopy as previously described [28]. The number of colonies was converted to the number of colonies per 10^5^ cells transplanted using the following equation:

$$Colonies∕10^{5}cells\;transplanted\;=\;number\;of\;colonies\;\times \;10^{5}∕number\;of\;cells\;transplanted$$

To account for the potential effects of the *n*-BuOH fraction of *P*. *japonicus* (PJB) on SSC proliferation, the total number of colonies was also calculated as the number of colonies per total cells recovered after culture using the following equation:

Relative number of recovered SSCs = (number of colonies∕number of cells transplanted) × total number of cells recovered

To determine the extent of donor spermatogenesis in the recipient testes, the testes were obtained three months after transplantation, cryosectioned, and visualized histologically using fluorescence microscopy.

### Statistical analysis

All statistical analyses were conducted using SPSS software (version 18; SPSS Inc., Mechanicsburg, PA, USA). Analysis of variance (ANOVA) and Tukey’s honestly significant difference (HSD) test were used to test for differences in viability, proliferation, and the number of colonies between treatment groups. Differences were considered significant at *p* values less than 0.05. Unless otherwise stated, all experiments were conducted in triplicate.

## Results

### Screening natural plant-derived extracts for SSC proliferation-promoting effects

We investigated the proliferation-promoting effects of various natural plant-derived extracts on SSCs cultured *in vitro*. In every SSC culture experiment, GDNF was used together with the extracts because GDNF is critical for mouse SSC self-renewal in a serum-free, chemically defined culture system. In these experiments, we searched for extracts that exerted a synergic effect with GDNF. Cells treated with GDNF alone served as the control group. To each of the five other SSC culture groups, GDNF and one of the five natural plant-derived extracts at three different concentrations (0.1, 1, and 10 μg/mL) were added to the culture medium. After seven days of culture, we looked for an increase, especially a dose-dependent increase, in the number of cells compared to that in the control group (Fig 1). A dose-dependent increase in the number of cells was observed in the *Chrysanthemum indicum-* and PJM-treated groups. However, only PJM treatment resulted in significantly higher cell proliferation (132.4 ± 5.6%) compared to the control treatment.

### Assessment of the proliferation-promoting effects of fractions from the *P*. *japonicus* extract on mouse SSCs cultured under serum-free conditions

**Fig 2:**
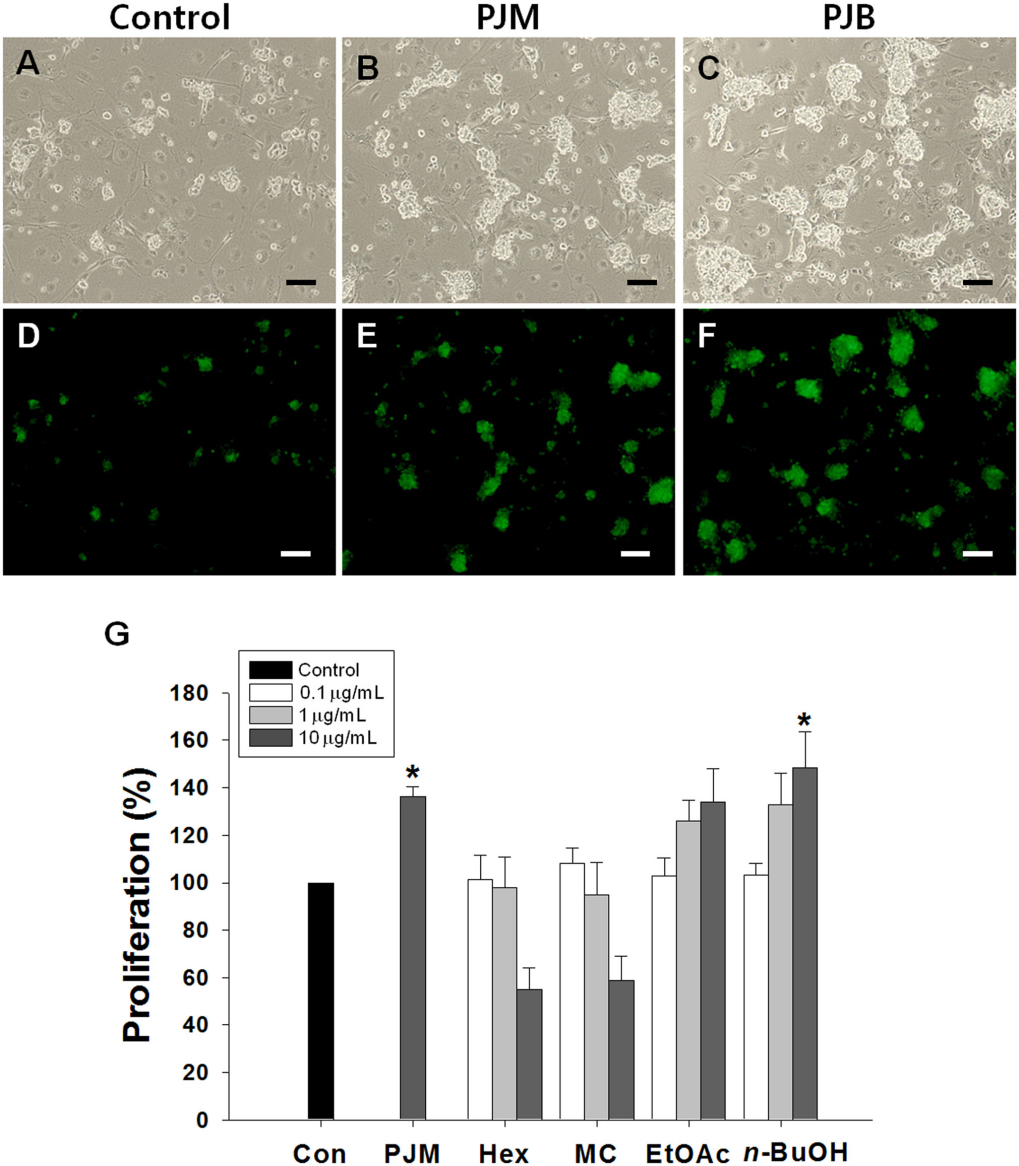
The effect of *Petasites japonicus* on SSC proliferation activity. (A) EGFP-positive germ cells enriched for SSCs were cultured for seven days in serum-free medium containing GDNF either without any plant extract (Control) or with PJM or PJB. The cells were examined by bright field (upper) and fluorescence (bottom) microscopy. Scale bars = 100 μm. (B) The proliferation-promoting effects of each *P*. *japonicus* fraction on SSCs cultured for seven days. Values are means ± SEM (n = 6 independently established cultures for each treatment). Con, control; PJM, *P*. *japonicus* extract; Hex, n-hexane fraction; MC, methyl chloride fraction; EtOAc, ethyl acetate fraction, n-BuOH, n-butanol fraction (PJB). Asterisks indicate a significant increase (*P* < 0.05) compared to that of the control.

We selected PJM for further fractionation with sequential extractions using *n*-hexane, MC, EtOAc, and *n*-BuOH to investigate the proliferation-promoting effects on SSCs. Each extracted fraction was added to serum-free culture medium at 0.1, 1, and 10 μg/mL, and the proliferation-promoting effects of the fractions were assessed by comparison to the proliferation of the control after seven days of culture. As shown in Fig 2B, the hexane and MC fractions decreased the proliferation of SSCs in a dose-dependent manner when compared to that of the control group. In contrast, there was a tendency toward a dose-dependent increase in the number of cells in both the EtOAc- and PJB-treated groups. However, only 10 μg/mL PJB had a significant effect on the proliferation of SSCs when compared to that of the control group (Fig 2B). In addition, in the PJB-treated group (10 μg/mL), we noted the formation of cell clumps, which is one of the characteristics of SSCs. These clumps expressed EGFP, which allowed for the identification of donor cells using fluorescence microscopy (Fig 2A; PJB). Furthermore, the size and number of the clumps in the PJB-treated group (10 μg/mL) were greater than those in the control group (Fig 2A). These results suggest that PJB might have proliferation-promoting effects on SSCs cultured *in vitro*.

### Characterization of SSCs cultured with PJB

**Fig 3:**
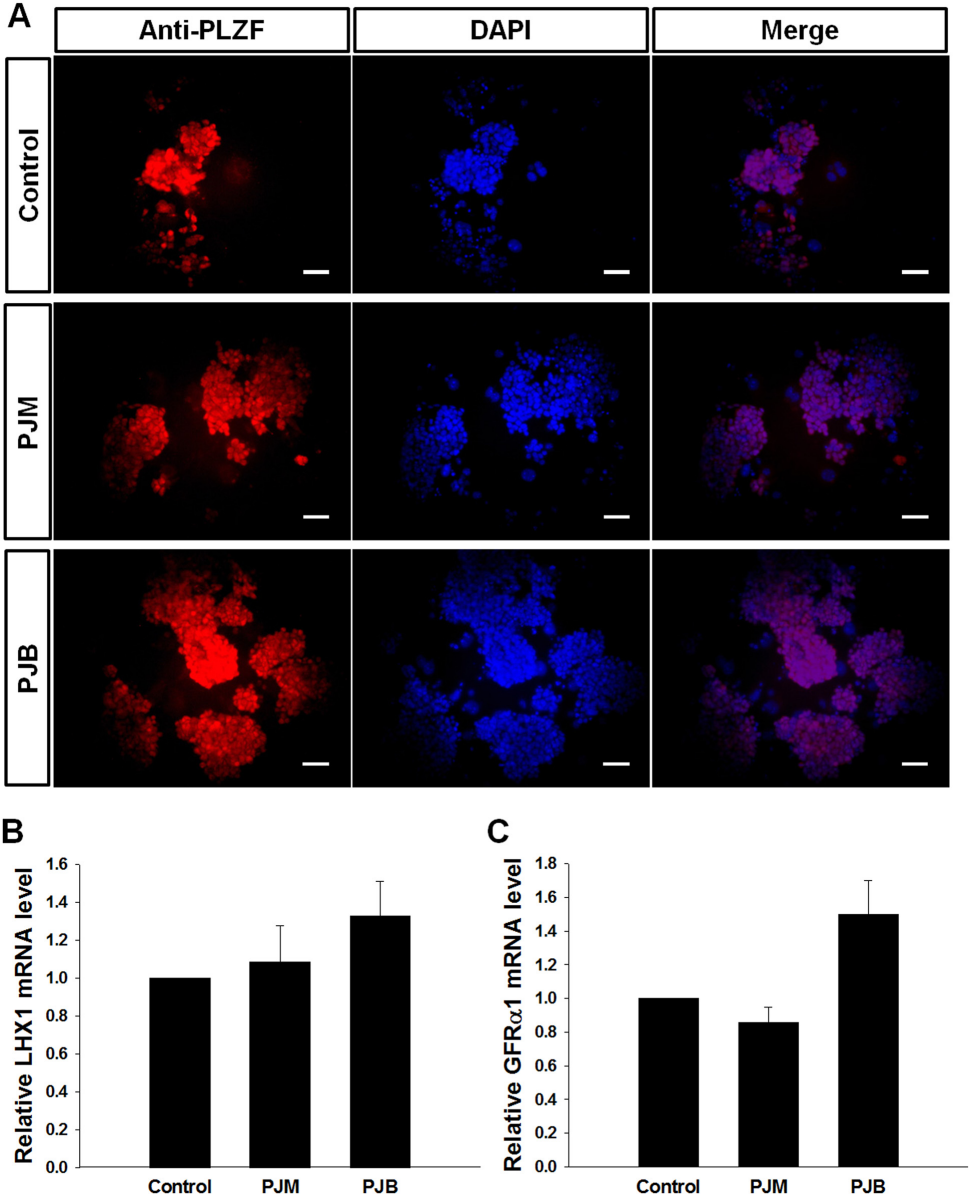
PLZF expression in germ cells cultured with PJB. (A) PLZF protein expression was assessed in undifferentiated germ cells cultured with only GDNF (Control), GDNF and 10 μg/mL PJM (PJM), or GDNF and 10 μg/mL PJB (PJB) under mSFM conditions by immunostaining. PLZF is a marker for undifferentiated spermatogonia, including SSCs. Scale bar = 100 μm. (B, C) Real-time PCR analysis of the expression of the undifferentiated spermatogonia or spermatogonial stem cell markers *LHX1* (B) and *GFRα1* (C) in germ cells cultured with only GDNF (Control), with GDNF and PJM, or with GDNF and PJB (Bars are the mean ± SEM; n = 3).

Treatment of SSCs with PJM or PJB at 10 μg/mL significantly increased proliferation compared with the proliferation of the control-treated cells. Therefore, we assumed that PJM and PJB had the strongest proliferation-promoting effects on SSCs and further investigated the characteristics of SSCs cultured with PJM or PJB using immunocytochemistry and qRT-PCR. To characterize the treated SSCs, germ cell clumps containing SSCs obtained after seven days of *in vitro* culture with and without extracts were analyzed by assessing the expression of PLZF by immunocytochemistry. We found that PLZF was normally expressed in the germ cell clumps from the PJM- and PJB-treated groups. Normal *PLZF* expression was also observed in the control group, although the size of the germ cell clump was smaller than that in the PJB-treated group (Fig 3A).

To determine the expression of *LIM homeobox 1* (*LHX1*) and *GFRα1* in the treatment groups, germ cells cultured with the extracts were analyzed using qRT-PCR. Although in the PJB-treated group, the relative expression of *LHX1* and *GFRα1* was 1.3 ± 0.2 and 1.5 ± 0.2 fold higher, respectively, than in the control group, these differences were not statistically significant (Fig 3B and 3C).

### The activity of SSCs cultured with PJB

**Fig 4:**
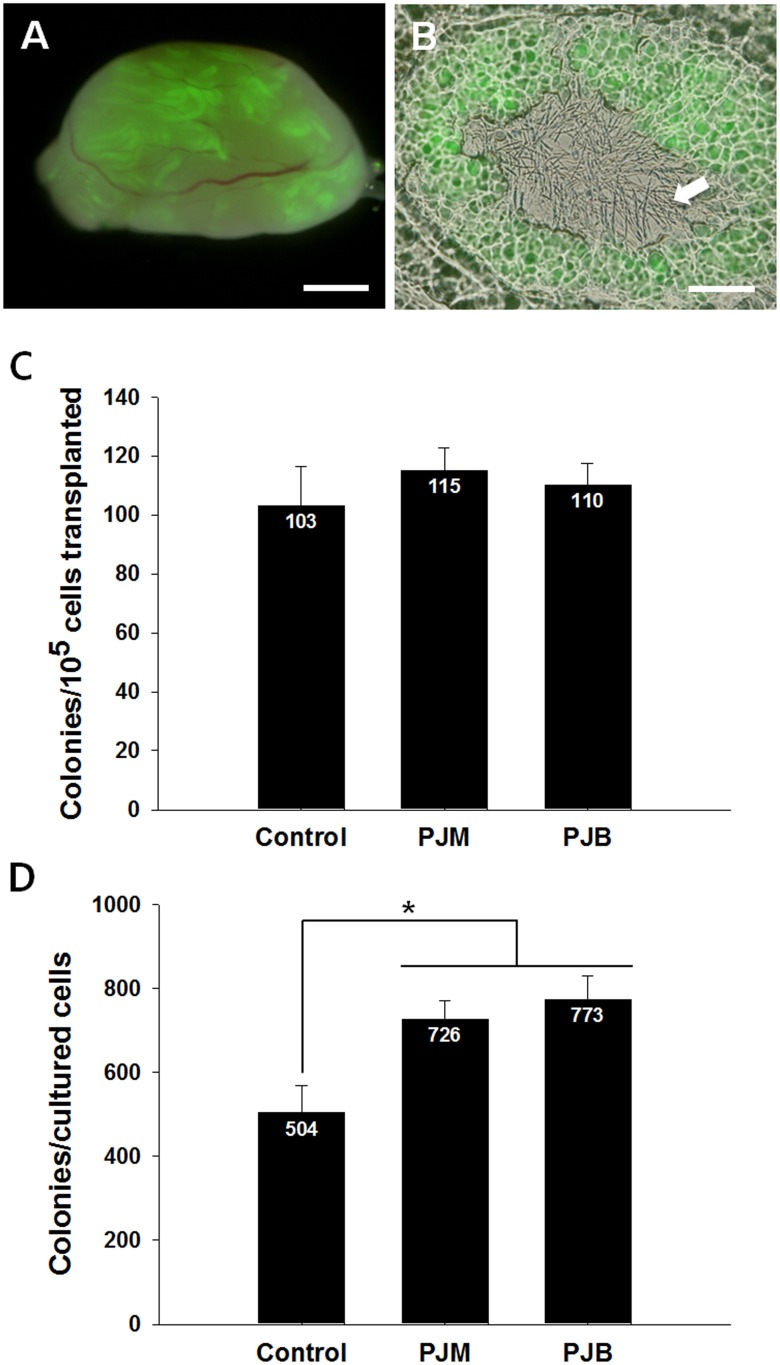
The effect of PJB on spermatogonial stem cell activity. Spermatogonial stem cell activity was assessed by counting the number of colonies derived from donor spermatogonial stem cells after transplantation. (A) Dark-field fluorescence images of a recipient testis transplanted with germ cells that were cultured with 10 μg/mL PJB. The donor spermatogenic colonies in the recipient seminiferous tubules are a distinct green color. (B) Cryosections of donor-derived germ cell colonies. Complete spermatogenesis was detected as the presence of sperm (white arrow) in the lumen of the seminiferous tubules. Scale bars: (A) = 2 mm; (B) = 50 μm. (C) The number of colonies per 10^5^ transplanted cells. (D) Relative number of SSCs recovered after culture with 10 μg/mL PJB. Values are mean ± SEM (n = 3 cultured and transplanted samples). The total number of mice/testes analyzed in the control (cultured without extract), PJM (cultured with 10 μg/mL PJM), and PJB (cultured with 10 μg/mL PJB) group was 5/9, 6/12, and 6/12, respectively. Asterisks indicate a significant difference (*P* < 0.05) compared to the control.

Our results on the proliferation of SSCs and the expression of undifferentiated germ cell markers showed that PJB might promote the proliferative activity of an undifferentiated germ cell population containing SSCs. However, further direct verification of the proliferation-promoting effect of PJB on SSCs was needed. A definitive method for assessing SSC activity in germ cells recovered after culture is the germ cell transplantation assay. Therefore, to evaluate the proliferation-promoting effect of the different extracts on SSCs, three groups of germ cells, we plated 2 × 10^5^ cells into a 24-well plate. After 1 week culture, the control (germ cells cultured without any plant extract), PJM (germ cells cultured with PJM; 10 μg/mL), and PJB (germ cells cultured with PJB; 10 μg/mL) groups were recovered, and we counted control (4.89 ± 0.11 × 10^5^), PJM (6.31 ± 0.11 × 10^5^), and PJB (7.03 ± 0.35 × 10^5^) treated cells. This data showed that the PJM and PJB treated cells were 3.2 and 3.5 fold higher, respectively, than the number of seeding cells, and PJM and PJB were significantly higher than control group. These cells were then transplanted into recipient mouse testes that were previously treated with busulfan to eliminate endogenous germ cells. Two months after transplantation, the recipient mice were sacrificed to count the number of GFP colonies derived from the donor germ cells. In the recipient testes, colonies expressing GFP were apparent under a fluorescence microscope, and it appeared that the GFP-expressing colonies were derived from the donor SSCs (Fig 4A). We also observed complete spermatogenesis in histological sections of the donor-derived colonies (Fig 4B). There was no significant difference in the number of colonies per 10^5^ transplanted cells between the control (103.1 ± 13.1 colonies), PJM (115.1 ± 7.6 colonies), and PJB (109.9 ± 7.4 colonies) groups, indicating that the ratio of SSCs in the cultures did not differ between treatment groups (Fig 4C). However, when the data were normalized to the total number of recovered cells after seven days of culture, the number of colonies in the control, PJM, and PJB groups was 504.3 ± 63.3, 726.0 ± 44.7, and 773.4 ± 55.6, respectively (Fig 4D). These results suggest that PJB can promote SSC proliferation activity *in vitro*.

## Discussion

To the best of our knowledge, our study is the first to report effective natural plant-derived extracts for stimulating SSC proliferation in culture. Our results show the potential of PJB for stimulating SSC proliferation *in vitro* (Fig 2). The undifferentiated nature of the germ cells following culture in the presence of PJB was supported by our examination of PLZF, LHX1, and GFRα1 expression (Fig 3). PLZF, LHX1, and GFRα1 are known to be primarily expressed in undifferentiated spermatogonia at the basement membrane of the seminiferous tubule. These proteins are essential for maintenance of the undifferentiated state of the cells. If the function of these proteins is lost, the balance between stem cell self-renewal and differentiation shifts from self-renewal toward differentiation [29–31]. Thus, normal expression of *PLZF*, *LHX1*, and *GFRα1* has been recognized as an important indicator of SSC function, including their self-renewal ability.

Although we confirmed the stimulating effect of PJB on SSCs based on proliferation data, the *in vitro* proliferation analysis did not directly quantify the effects of this fraction on SSC function. However, culture conditions affecting SSC functionality can be unequivocally evaluated by a transplantation assay. Two months after transplantation, no difference was observed in the number of colonies per 10^5^ transplanted cells between the groups, indicating that the cultured SSCs were functionally normal and were not adversely affected by the PJB (Fig 4C). However, it was difficult to elucidate the differences in the SSC proliferation rate between treatment groups. Conversion of the number of colonies per 10^5^ transplanted cells to relative number of recovered SSCs showed that the SSCs in the PJB group generated significantly more colonies than the control group did, demonstrating that PJB increased the total number of SSCs in the germ cell populations compared to that in the control group (Fig 4D). Additionally, sperm derived from the transplanted SSCs were observed within the seminiferous tubule (Fig 4B), indicating that complete spermatogenesis of the donor SSCs occurred.

Overall, our data, which were derived from a combination of functional transplantation experiments, proliferation assays, and gene expression analysis, demonstrate that not only PJM but also PJB promote the proliferation of SSCs *in vitro*. *P*. *japonicus*, which belongs to the Compositae family, is mainly distributed and cultivated as a perennial herbaceous plant in Eastern Asian countries, including Korea, Japan, and Taiwan [32]. The petioles, rhizomes, and whole plant of *P*. *japonicus* have not only been consumed as a food in Asian countries, but have also been used for therapeutic purposes in European countries [32,33]. Given these findings, PJB might affect the proliferation of SSCs by reducing oxidative stress through its antioxidant effects in culture. Oxidative stress is a phenomenon caused by oxidation generated during the metabolism of intracellular lipids, proteins, carbohydrates, and nucleic acids. If the oxidation is abnormally high, mitochondria, the nucleus, structural and cytoplasmic proteins, and complex carbohydrates in cells can become damaged owing to the generation of excessive reactive oxygen species [34]. Oxidative stress may ultimately lead to serious damage to organs such as the testes, which contain metabolically active cells that undergo proliferation. Thus, maintenance of intracellular antioxidant activity plays a very important role throughout life in males because oxidative stress could inhibit male reproductive function [35]. PJM has been reported to be effective as a relaxant and against stomach cramps, asthma, cough, and amenorrhea, and its analgesic effect is widely applied for the treatment of headaches [36]. Thus far, no side effects have been reported with the use of PJB, which could be an indication of its pharmacological applications. Further in-depth studies investigating the factors that are directly related to the antioxidant effect of *P*. *japonicus* on SSCs at the molecular level are needed. In addition, there is a need for substances that could be used as safe traditional medicines or as medicinal products that are expected to be potentially useful for the treatment and prevention of male infertility.

## Supporting Information

S1 Checklist Completed “The ARRIVE Guidelines Checklist” for reporting animal data in this manuscript. (PDF)
