# Correction: *Petasites japonicus* Stimulates the Proliferation of Mouse Spermatogonial Stem Cells

**DOI:** 10.1371/journal.pone.0144480

**Published:** 2015-12-02

**Authors:** Hye-Ryun Kang, Yong-An Lee, Yong-Hee Kim, Dong Gu Lee, Bang-Jin Kim, Ki-Jung Kim, Byung-Gak Kim, Myeong-Geun Oh, Chan Kyu Han, Sanghyun Lee, Buom-Yong Ryu

There is an error in affiliation 3 for author Byung-Gak Kim. Affiliation 3 should be: Department of Obstetrics, Gynecology & Reproductive Biology, Michigan State University, Grand Rapids, Michigan, United States of America.
